# Concurrent Encounter of Superior Mesenteric Artery Syndrome and Nutcracker Syndrome in a Young Female Patient: A Case Report and Literature Review

**DOI:** 10.7759/cureus.66002

**Published:** 2024-08-02

**Authors:** Abdullah Almunifi, Abdullah Z Al-Dhayan, Musab Alanazi, Omar A Ababtain

**Affiliations:** 1 Department of Surgery, College of Medicine, Majmaah University, Majmaah, SAU; 2 Department of Surgery, Prince Mohammed Bin Abdulaziz Hospital, Riyadh, SAU; 3 Department of Surgery, College of Medicine, King Saud University Medical City (KSUMC), King Saud University, Riyadh, SAU

**Keywords:** wilkie syndrome, superior mesenteric artery syndrome, renal nutcracker syndrome, left renal vein, case report, rare diseases

## Abstract

Superior mesenteric artery (SMA) syndrome causes duodenal obstruction between the SMA and aorta, which culminates into bowel obstruction. Meanwhile, nutcracker syndrome (NCS) involves left renal vein compression between the aorta and SMA, categorized by the compression site. We present a 15-year-old female with no prior medical or surgical history who displayed early signs of the rarely coexisting SMA and nutcracker phenomena, which were managed symptomatically along with nutritional support to reach her optimal body mass index.

## Introduction

Superior mesenteric artery (SMA) syndrome, also known as Wilkie’s syndrome, chronic duodenal ileus, or cast syndrome, refers to the entrapment and obstruction of the third part of the duodenum between the SMA and the aorta [1]. The classical symptoms of this condition may include chronic postprandial nausea, vomiting, anorexia, weight loss, esophageal reflux, and epigastric abdominal pain. Gastroscopy, including biopsy and contrast imaging, alongside complementary radiological studies, constitutes pivotal diagnostic avenues in the pursuit of identifying SMA syndrome. To mitigate the sequelae of bowel obstruction, management of SMA syndrome varies from conservative nutritional approaches to surgical interventions [2].

The nutcracker syndrome (NCS), or left renal vein (LRV) entrapment syndrome, denotes the compression of the LRV, predominantly situated between the abdominal aorta and the SMA. It is categorized into pre-aortic compression and post-aortic compression depending on the site of the compression [3,4]. This rare condition, often associated with anatomical variations, typically presents with flank pain, occasionally accompanied by macroscopic or microscopic hematuria, proteinuria, or renal insufficiency [3,5]. As such, the diagnosis of NCS is established through a combination of clinical assessment and radiological evidence [6]. The management of NCS is based on clinical presentation and LRV hypertension severity, with treatment options ranging from surveillance to nephrectomy, guided by symptom severity and potential reversibility [6,7].

To date, due to the scarcity of documented cases detailing distinct clinical presentations in such scenarios, we present a case report involving a juvenile female patient from Saudi Arabia, demonstrating the simultaneous manifestation of NCS and Wilkie's syndrome. This case has been meticulously documented in accordance with the most recent standardized Surgical CAse REport (SCARE) criteria, showcasing a commitment to rigorous academic protocols [8].

## Case presentation

We present a 15-year-old female patient with no significant medical or surgical history who presented to the emergency department with a one-day history of abrupt and intense compressive pain localized to the epigastric region. The pain was non-radiating and accompanied by nausea and repeated episodes of vomiting food contents. Symptoms were aggravated with oral intake but relieved with fasting and analgesia. Additionally, the patient reported unintentional weight loss from 53 kg to 42 kg over several months, attributed to decreased appetite and stress.

**Figure 1:**
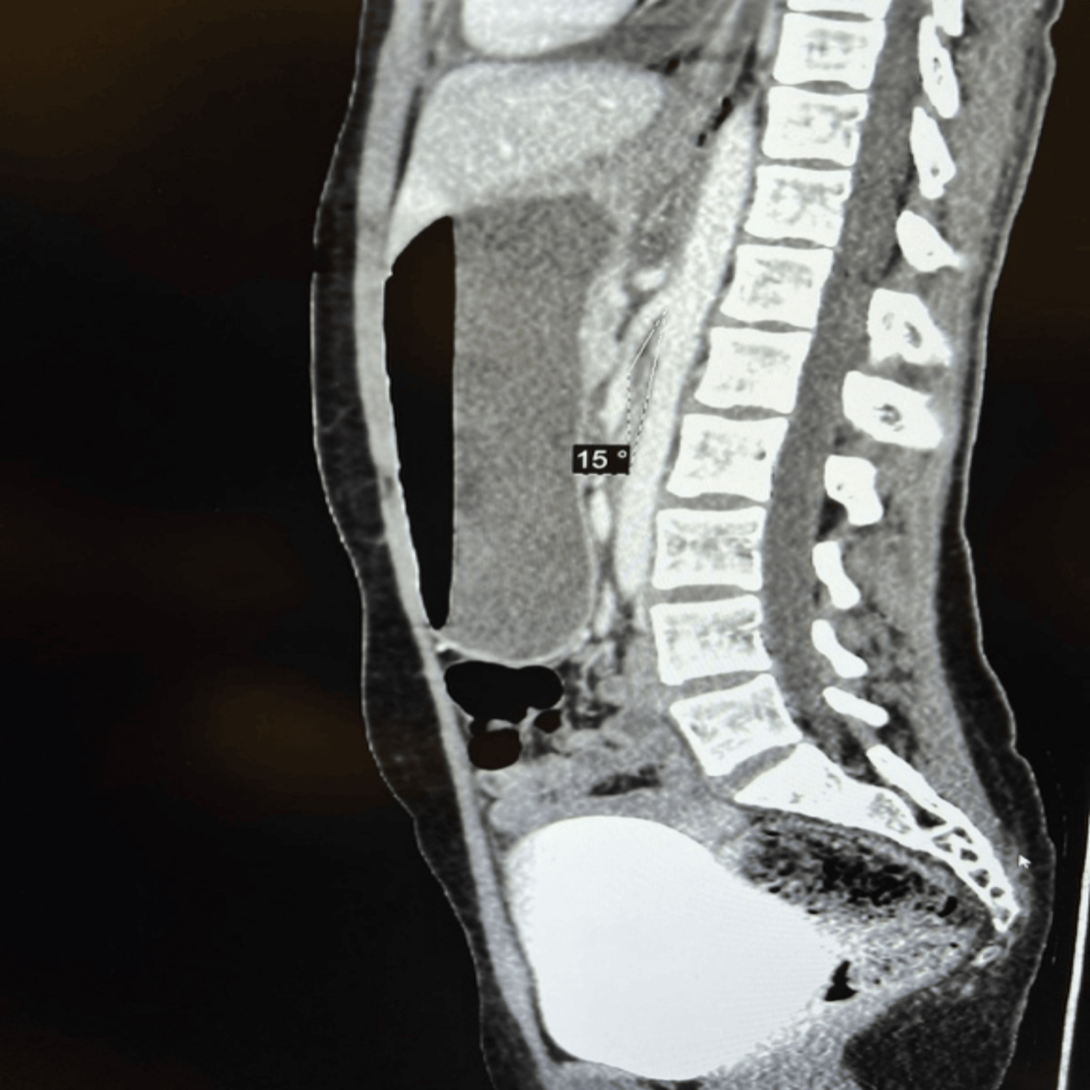
CT sagittal section demonstrating an aortomesenteric angle of 15°. CT: computed tomography.

**Figure 2:**
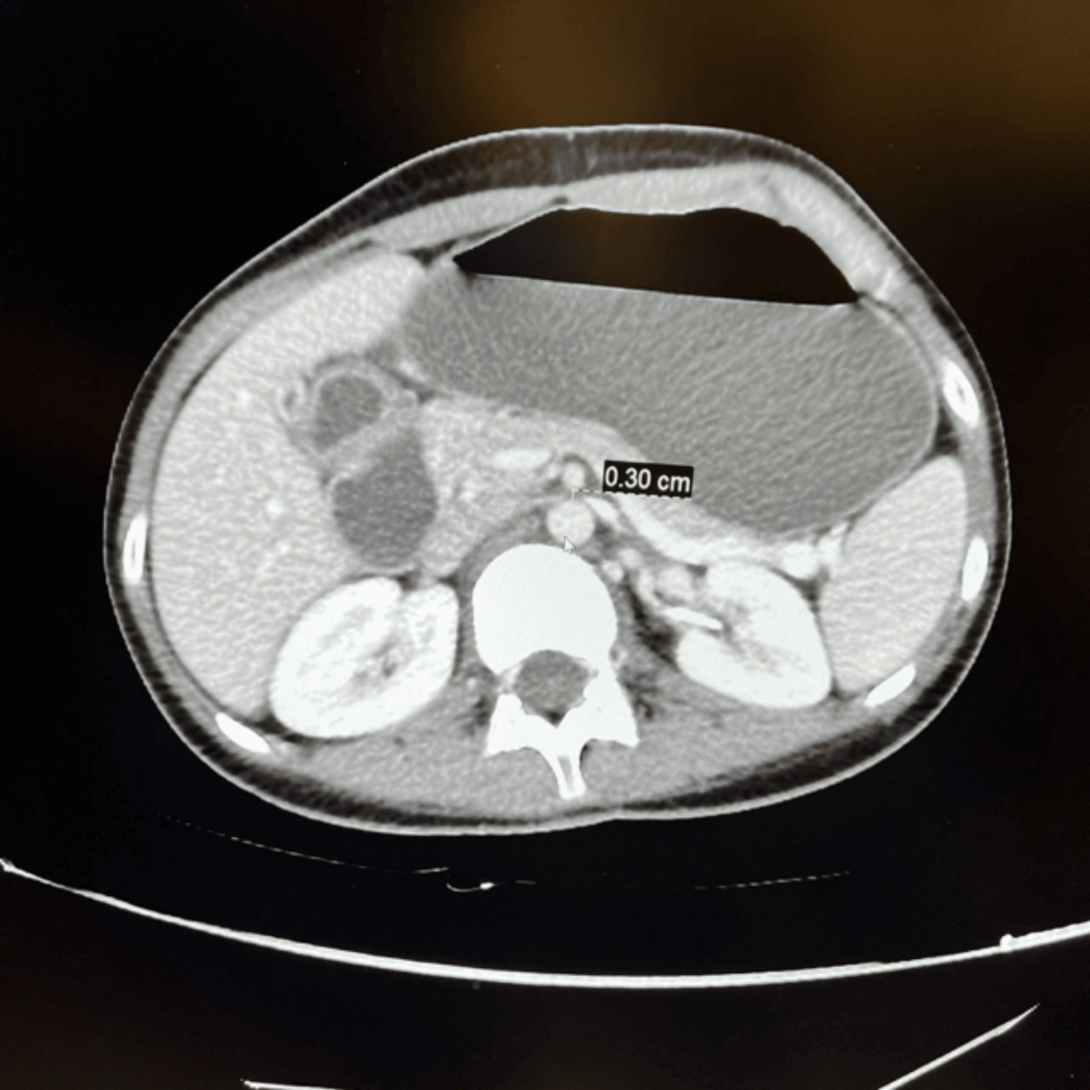
CT axial section showing aortomesenteric distance of 30 mm. CT: computed tomography.

A computed tomography (CT) scan revealed a significantly dilated stomach along with the first, second, and subsequent portions of the duodenum. This was correlated with a decreased aortomesenteric angle of 15° and distance of 30 mm, indicating SMA, and a dilated LRV proximal to the constriction between the SMA and the aorta, suggestive of NCS (Figures 1, 2).

After a normal upper endoscopy finding, with passage into the second part of the duodenum, the patient was admitted and received intravenous (IV) fluids, analgesia, and antiemetic medications. Consequently, she was kept on total parenteral nutrition (TPN) for nine days and then started on oral high-calorie nutritional supplements called SureNutri Hi Calorie. Each 200 ml serving of SureNutri Hi Calorie provides 300 kcal of energy, 11.4 g of protein (15% of the composition), 43 g of carbohydrates (57% of the composition), and 9.16 g of total fats (28% of the composition). The patient was able to tolerate the oral supplements on the second day after starting TPN. On admission, her weight was 41 kg, which improved to 48 kg upon discharge and to about 55 kg during her follow-up visit at the clinic, showcasing a body mass index (BMI) of 23, which is an ideal body mass for her.

## Discussion

SMA syndrome

First described by Carl von Rokitansky in 1842, the compression of the SMA has been known by different names over time. Eventually, Georg A. Keisler coined the term "Superior Mesenteric Artery (SMA) Syndrome" to describe this condition in academic discussions, along with Wilkie's syndrome, named after David Wilkie, who described it comprehensively [9].

Epidemiology

The prevalence of this disorder spans from about 0.013% to 0.3% across the general population, with predisposing factors encompassing recent weight reduction, eating irregularities, dietary practices, post-scoliosis surgical interventions, and burn trauma. Predominantly afflicting females, the condition typically emerges within the age bracket of 10-39 years [10,11].

Etiologies and pathophysiology

SMA syndrome arises from a spectrum of etiologies linked to weight loss, malabsorption, malignancy, acquired immunodeficiencies, bariatric surgery, burns, spinal cord injury, paraplegia, prolonged bed rest drug abuse, anorexia nervosa, and thyrotoxicosis [12]. The pathophysiological cascade is characterized by the loss of mesenteric fat planes, a pivotal mechanism that reduces cushioning capacity and intensifying vulnerability to vascular compression [10,11].

Radiological diagnosis of SMA

Radiological findings are pivotal in confirming the diagnosis of SMA syndrome, employing modalities such as CT, magnetic resonance (MR) angiography, and ultrasonography. Normal anatomical measurements encompass an SMA-aorta angle of 38-65° and an aortomesenteric distance of 10-34 mm. Anatomical anomalies contributing to SMA syndrome involve a short suspensory ligament of Treitz, leading to elevated duodenojejunal flexure suspension, malrotation of the intestine, and increased lumbar lordosis. External factors, such as body casting pressure, may also contribute. Therefore, radiological-specific criteria for SMA syndrome include duodenal obstruction, an aortomesenteric artery angle ≤25°, aortomesenteric distance ≤8 mm, and abnormal duodenal fixation by the ligament of Treitz or a low SMA origin [10,11,13].

Nutcracker syndrome

The nutcracker anatomy often manifests asymptomatically, underscoring the importance of imaging for its detection [14]. Similarly, due to the pathophysiologic vascular compression between the SMA and NCS, diagnosing both syndromes hinges on radiological evidence showing obstruction of the horizontal third part of the duodenum (D3), along with a reduced angle between the aorta and SMA and a narrowed aortomesenteric angle [2,15].

Anatomical types

NCS showcased distinct anatomical manifestations in vascular compression and imaging characteristics, notably featuring constriction of the LRV by the traversing vertical SMA, culminating in hindered venous outflow (anterior nutcracker). Furthermore, this syndrome presents a distinct variant where compression occurs between the aorta and vertebral bodies, known as the retro-aortic LRV (posterior nutcracker) [15]. Hence, this clarifies the observed presentations in patients. In rare instances, left splenorenal shunts may develop due to extensive proximal stenosis of the LRV. These shunts also serve as collateral circulation to the left gonadal vein [5].

The compression ratio (CR), which indicates the severity of venous compression, is calculated by dividing the length of the pre-compressed segment by that of the compressed segment. A ratio of the LRV over 2.25 demonstrated 91% specificity and sensitivity for NCS [16].

Management of SMA

The objective of managing SMA syndrome is to replenish the mesenteric fat pad between the aorta and SMA, with a target to widen the angle between these vessels and alleviate obstruction. Guidelines from the National Institute for Health and Care Excellence for evaluating nutritional status in adults receiving enteric nutrition and TPN can be employed (Table 1). Surgical interventions for SMA syndrome, such as feeding jejunostomy, Strong's procedure, gastrojejunostomy, or duodenojejunostomy, may also be considered, particularly in patients with inadequate response or unfeasibility for oral feeding or TPN [17].

**Table 1:** TPN components. TPN: total parenteral nutrition.

TPN components	
Solution (volume)	2,100 mL
Dextrose (grams)	312.5 g/day
Dextrose (percent)	50%
Dextrose calculation from percent	625 mL
Protein (volume)	500 mL
Protein (percent)	15%
Protein (grams)	75 g/day
Fat (mL)	100 mL
Fat (grams)	20 g/day
Lipid (percent)	20%
Dextrose total kcal calculation from gram calculation	1,063 kcal
Protein concentrate total kcal calculation	300 kcal
Total lipid kcal calculation	180 kcal
KCl 15% amount	40 mmol/day
KCl 15% volume calculation	20 mL
NaPO_4_ Glycophos amount	20 mmol/day
NaPO_4_ Glycophos volume calculation	20 mL
MgSO_4_ 10% amount	4 mmol/day
MgSO_4_ 10% volume calculation	10 mL
Ca gluconate amount	2.3 mmol/day
Ca gluconate volume calculation	10 mL
Trace elements	10

## Conclusions

In general, SMA syndrome and NCS are considered rare disorders; also, their co-occurrence is extremely rare and has been reported in only a few cases. Additionally, both share a common mechanism by compression of the third portion of the duodenum between the SMA and the aorta causing abdominal symptoms and compression of the LRV between the SMA and the aorta. Suspicion increased in patients who present to the ED with sudden abdominal pain associated with or without nausea or vomiting after remarkable weight loss. We highlight the importance of the combination therapy of long-term nutritional support, including TPN and high-caloric diet, once the patient started to tolerate orally with dietician involvement and follow-up.
